# Characterization of Genes Encoding Key Enzymes Involved in Anthocyanin Metabolism of Kiwifruit during Storage Period

**DOI:** 10.3389/fpls.2017.00341

**Published:** 2017-03-10

**Authors:** Boqiang Li, Yongxiu Xia, Yuying Wang, Guozheng Qin, Shiping Tian

**Affiliations:** ^1^Key Laboratory of Plant Resources, Institute of Botany, Chinese Academy of SciencesBeijing, China; ^2^College of Life Sciences, University of Chinese Academy of SciencesBeijing, China

**Keywords:** red fleshed kiwifruit, anthocyanin, low temperature, molecular basis, MYB transcription factor

## Abstract

‘Hongyang’ is a red fleshed kiwifruit with high anthocyanin content. In this study, we mainly investigated effects of different temperatures (25 and 0°C) on anthocyanin biosynthesis in harvested kiwifruit, and characterized the genes encoding key enzymes involved in anthocyanin metabolism, as well as evaluated the mode of the action, by which low temperature regulates anthocyanin accumulation in ‘Hongyang’ kiwifruit during storage period. The results showed that low temperature could effectively enhance the anthocyanin accumulation of kiwifruit in the end of storage period (90 days), which related to the increase in mRNA levels of *ANS1, ANS2, DRF1, DRF2*, and *UGFT2*. Moreover, the transcript abundance of *MYBA1-1* and *MYB5-1*, the genes encoding an important component of MYB–bHLH–WD40 (MBW) complex, was up-regulated, possibly contributing to the induction of specific anthocyanin biosynthesis genes under the low temperature. To further investigate the roles of AcMYB5-1/5-2/A1-1 in regulation of anthocyanin biosynthesis, genes encoding the three transcription factors were transiently transformed in *Nicotiana benthamiana* leaves. Overexpression of AcMYB5-1/5-2/A1-1 activated the gene expression of *NtANS* and *NtDFR* in tobacco. Our results suggested that low temperature storage could stimulate the anthocyanin accumulation in harvested kiwifruit via regulating several structural and regulatory genes involved in anthocyanin biosynthesis.

## Introduction

Anthocyanins are one of the most important plant pigments and usually accumulate in specific plant tissues, such as leaves, roots, fruits, and flowers, contributing to their red, blue, purple and dark color. As an important secondary plant metabolite, anthocyanins have many functions in plants, ranging from the resistance to UV, light and pathogen to the attraction of pollinators and seed dispersers for reproduction (Ubi et al., 2006; Jaakola, 2013). Moreover, anthocyanins have been extensively used in food to improve human health because of their specific function in antioxidant activity, such as prevention of heart disease and anticancer activity (He and Giusti, 2010). Fruits and vegetables are still the common food source of anthocyanins due to the low stability of anthocyanins during processing in food system (Giusti and Wrolstad, 2003). However, poor ripening quality along with less accumulation of anthocyanins is always accompanied with fruit that were commercially harvested at ‘mature’ stage. In addition, some fruits, such as apple and pears, easily lose pre-harvest colors during inappropriate storage condition due to decreased ability to accumulate anthocyanin (Steyn et al., 2004). Therefore, exploring the regulating mechanism of anthocyanins biosynthesis in fruits has biologically interesting and economically significance.

Some genes involved in the anthocyanin pathway have been cloned and characterized in fruits (Montefiori et al., 2011; Tang et al., 2016). Among them, three key enzymes, dihydroflavonol 4-reductase (DFR), anthocyanidin synthase (ANS) and UDP-glucose:flavonoid 3-O-glucosyltransferase (UFGT or F3GT), contribute to the last step of anthocyanin biosynthesis, from the anthocyanidins to the anthocyanin (Jaakola, 2013). And these genes usually show different expression patterns in different fruits during the process of anthocyanin biosynthesis. For example, UFGT has been reported to be one of the mayor control points of anthocyanin biosynthesis in grape and litchi (Boss et al., 1996; Zhao et al., 2012), while the expression of DFR was found to be closely correlated with anthocyanin content in different genotypes of pomegranate (Wang et al., 2012). Moreover, anthocyanin pigment is also regulated by developmental factors and various environmental factors in plants. MYB–bHLH–WD40 (MBW) complex plays important role in regulation of anthocyanin synthesis in plants (Petroni and Tonelli, 2011). Among the three components, the R2R3 MYBs, which are the main secondary metabolism regulators, determine the pattering and spatial localization of anthocyanins (Liu et al., 2015). Many MYB genes involved in anthocyanin regulation are characterized in various fruits and flowers, such as apple, grape, blueberry, strawberry, cherry, and petunia (Jaakola, 2013; Cavallini et al., 2014). Additionally, light and temperature, serve as important environmental factors, have been reported to control anthocyanin biosynthesis in plant, particularly low temperature (Jaakola, 2013; Liu et al., 2015).

‘Hongyang’ kiwifruit is one of the most popular cultivars in China because of its special quality, including unique anthocyanin accumulation, high sugar and vitamin C (Wu et al., 2013). ‘Hongyang’ kiwifruit grown in high altitude with low temperature usually have the higher accumulation of anthocyanin during fruit development, than that grown in low altitude area (Man et al., 2015b). In the previous study, we compared the expression profiles of 25 oxidative stress-related genes in ‘Hongyang’ kiwifruit stored in air and CA by quantitative real-time polymerase chain reaction (qRT-PCR), and proved expression of *SOD3, CAT1, APX1, APX2* and *GR3* may predominantly contribute to the maintaining of antioxidative systems (Xia et al., 2016). In this study, we determined the changes of anthocyanin content in kiwifruit stored at room- and low-temperature, then cloned and characterized several structural and regulatory genes involved in anthocyanin biosynthesis in ‘Hongyang’ kiwifruit. Our findings provide evidence for understanding the effect of low temperature on anthocyanin biosynthesis in kiwifruit during post-harvest storage periods.

## Materials and Methods

### Fruit Material

Kiwifruit (*Actinidia chinensis* cv. Hongyang) were harvested from a commercial orchard in Dujiangyan City (Sichuan, China) at a commercial mature stage. Fruits of similar size and the absence of physical injuries or decays were selected, and randomly divided into 6 lots with 100 fruits each. Three lots were stored at low temperature (0°C) for 3 months (90 days), and other three were kept at room temperature (25°C) for 9 days. All fruits were enclosed with polyethylene film bag (0.02-mm thickness) to maintain relative humidity of about 95%. As replicates for each treatment (room- and cold-storage), three lots of 15 fruits were sampled in every interval of 3 or 30 days storage at room- or low-temperature, respectively. Fruit firmness and the soluble solids content (SSC) were measured as described (Xia et al., 2016). The kiwifruit sampled at the paired time points (3 days at 25°C vs. 30 days at 0°C, 6 days at 25°C vs. 60 days at 0°C, 9 days at 25°C vs. 90 days at 0°C) showed similar SSC and firmness. For further molecular and biochemical analysis, inner pericarp tissue collected from the equatorial region (1.5-cm thickness) of each fruit was cut into pieces, frozen in liquid nitrogen and stored at -80°C.

### LC-MS Analysis of Anthocyanin

Anthocyanin was extracted and analyzed according to the following procedure. Briefly, 10 g of frozen samples were ground and extracted in 30 ml of methanol containing 2% (v/v) formic acid. After vortex, the homogenates were sonic-treated in ice-cold water for 20 min and then inoculated at 4°C overnight, followed by centrifugation at 3000*g* for 10 min. The extract was then filtered through 0.22 μm polytetrafluoroethylene filters and retained for component analysis. The anthocyanin analysis was performed on an Agilent 1290 Infinity UHPLC system coupled to Agilent 6540 UHD Accurate-Mass Q-TOF mass spectrometer (Agilent Technologies, USA). A 10 μl aliquot of sample was injected on C18 (150 mm × 4.6 mm) column and separations were carried out using a binary solvent system of Solvent A (water +0.1% formic acid) and Solvent B (acetonitrile +0.1% formic acid) at a flow of 300 μl min^-1^. The initial mobile phase was 95%A 5%B, increased linearly to 30%A 70% B in 20 min, and held for 5 min before resetting to 95%A 5%B ready for the next injection. The column temperature was set to 30°C and anthocyanins were monitored at 520 nm. Quantification of anthocyanins was conducted by comparison with authenticated standard solution of cyanidin 3-O-galactoside (Sigma). Anthocyanin peaks were further identified by ESI-MS and the spectral data were obtained in positive ion mode over the range m/z 100–1000. The ESI voltage, capillary temperature, and capillary voltages was 39 V, 300°C and 7 μV, respectively.

### Gene Identification and Alignment

*Dihydroflavonol 4-reductase, ANS*, and *UDP-flavonoid 3-O-galactosyl transferase* (*UFGT*) genes from *A*. *chinensis* ‘Hongyang’ were initially cloned using gene specific primers (Supplementary Table S1) designed on *DFR* (EST FG410069.1), *ANS* (EST FG407400.1) and *UFGT* (EST FG405592.1) sequences from an available kiwifruit EST library (Crowhurst et al., 2008). Full-length cDNA were cloned using a Rapid-amplification of cDNA Ends (RACE) kit (Takara). Putative MYB transcription factors were identified from the kiwifruit genome database (Huang et al., 2013), and four full-length *MYB* genes highly expressed in fruit tissue were selected. The open reading frames (ORFs) were predicted using GENSCAN, and protein sequences were aligned using ClustalX2.1. Phylogenetic tree of MYB proteins were constructed by neighbor-joining matrix with 1,000 bootstrap replicates using MEGA6. Names of all cloned genes in this study were according to the previous report by Li et al. (2015), except *MYB4a* which was named according to the phylogenic analysis.

### RNA Extraction and Gene Expression Analysis

Total RNA was extracted according to the methods reported by Zhu et al. (2013) from 1 g of kiwifruit tissues. Using a Takara PrimeScript^®^ RT reagent kit with gDNA eraser, the first strand cDNA was synthesized by reverse transcription following manufacturer’s instruction. Quantitative real-time PCR was performed on StepOnePlus Real-Time PCR System (Applied Biosystems) using SYBR^®^Premix Ex Taq^TM^ (TliRNaseH Plus) (Takara, Japan). Gene-specific primers were designed with Primer Express software 3.0 and presented in Supplementary Table S2. The PCR program was conducted as follows: 15 s at 95°C; 40 cycles of 5 s at 95°C, and 30 s at 60°C; followed by an automatic melting curve analysis. Three independent biological replicates were measured for each sample. The relative expression level for each gene was calculated using the comparative Ct method (2^-ΔCt^ method) with a kiwifruit *actin* as a reference.

### Transient Expression in *Nicotiana benthamiana*

The CDS of AcMYB5-1, AcMYB5-2, and AcMYBA1-1 were amplified using specific primers (Supplementary Table S3) containing *Kpn*I and *BamH*I restriction enzymatic site, respectively. The PCR product was recombined with the linearized vector pCAMBIA2300 (In-Fusion HD Cloning Kit; Clontech). The resulting construct were sequenced and introduced into *Agrobacterium tumefaciens* strain GV3101. *Agrobacterium* were cultured on LB agar plates with kanamycin and incubated at 28°C. The freshly grown *Agrobacterium* were sedimented by centrifugation for 5 min at 6000 *g*, resuspended in infiltration buffer, and incubated at room temperature for 2 h before infiltration. Tobacco plants were grown for five to six weeks and the young leaves were syringe-infiltrated with *A. tumefaciens* suspension in abaxial side of the leaf. Control was infiltrated with *Agrobacterium* containing pCAMBIA2300 (empty vector) at the same time and the transient expression was assayed 5 days after infiltration.

### Statistical Analysis

All statistical analyses were carried out using SPSS 13.0 software (SPSS Inc., USA). Data from assay of anthocyanin content or gene expression were analyzed by one-way analysis of variance (ANOVA), and the mean comparison was performed by Duncan’s multiple range tests. Differences at *P* < 0.05 were considered as significant.

## Results

### Anthocyanin Profile

Red-flesh is the distinguishing feature of ‘Hongyang’ kiwifruit due to the accumulation of anthocyanin in its inner pericarp during ripening. To identify the main anthocyanin in kiwifruit during storage, UHPLC Q-TOF-MS was applied to analyze the anthocyanin composition from the extract of fruit. Our results showed that all samples of ‘Hongyang’ kiwifruit presented two peaks that were identified as cyanidin 3-O-xylo(1-2)-galactoside and 3-O-galactoside by comparing both retention times and ESI-MS data (Supplementary Figure S1). The dominant anthocyanin peak had a molecular ion of m/z 581, while another peak had a molecular ion of m/z 499. The results indicated that cyanidin 3-O-xylo(1-2)-galactoside was the major anthocyanin present in ‘Hongyang’ fruit during storage, which is consistent with previous report in pre-harvest ‘Hongyang’ fruit (Man et al., 2015b).

### Effect of Low Temperature Storage on Fruit Color, Anthocyanin Content, and Fruit Quality

**FIGURE 1:**
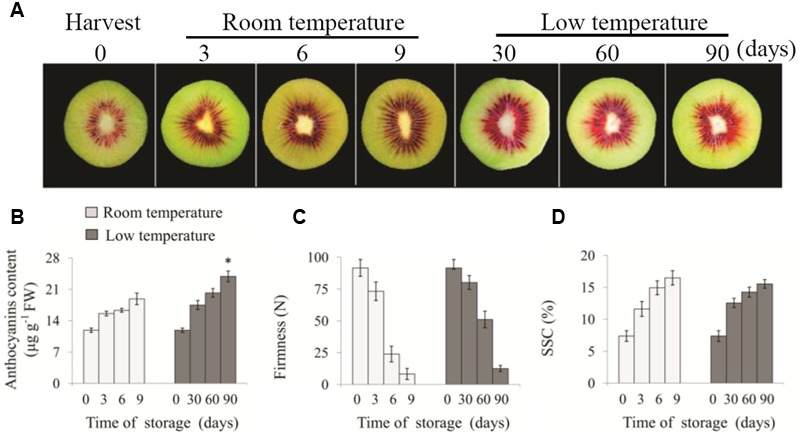
**Changes of appearance (A)**, anthocyanin content **(B)**, firmness **(C)** and SSC **(D)** in ‘Hongyang’ kiwifruit during storage at room temperature for 9 days or low temperature for 90 days, respectively. Data are means ± SD of three replicates. Error bars represent standard error of means. Asterisk indicates significant differences between fruits stored at room temperature and low temperature at the end of storage according to the Student’s *t*-test at *P* < 0.05.

As shown in **Figure 1A**, after 90 days of storage at low temperature, the red coloration of fruit inner pericarp was more notable than that of fruit at room temperature for 9 days. To investigate the influence of low temperature on anthocyanin content in ‘Hongyang’ fruit, the accumulation of major anthocyanin during the storage was investigated. The 3-O-xylo(1-2)-galactoside content in inner pericarp increased gradually with prolonged storage time (**Figure 1B**). In room temperature-stored fruit, it increased rapidly from 11.88 at 0 day to 18.87 μg⋅g^-1^ FW at 9 days. By contrast, in low temperature-stored fruit, it accumulated gradually and reached a peak value of 23.87 μg⋅g^-1^ FW after 90 days. At the same time, fruit firmness decreased obviously with storage time prolonging (**Figure 1C**), and SSC increased gradually (**Figure 1D**) in kiwifruit both stored at 25 and 0°C. The firmness and SSC of fruit stored at room temperature for 9 days and low temperature for 90 days showed no significant difference.

### Effect of Low Temperature Storage on the Expression of Anthocyanin Biosynthesis Genes

**FIGURE 2:**
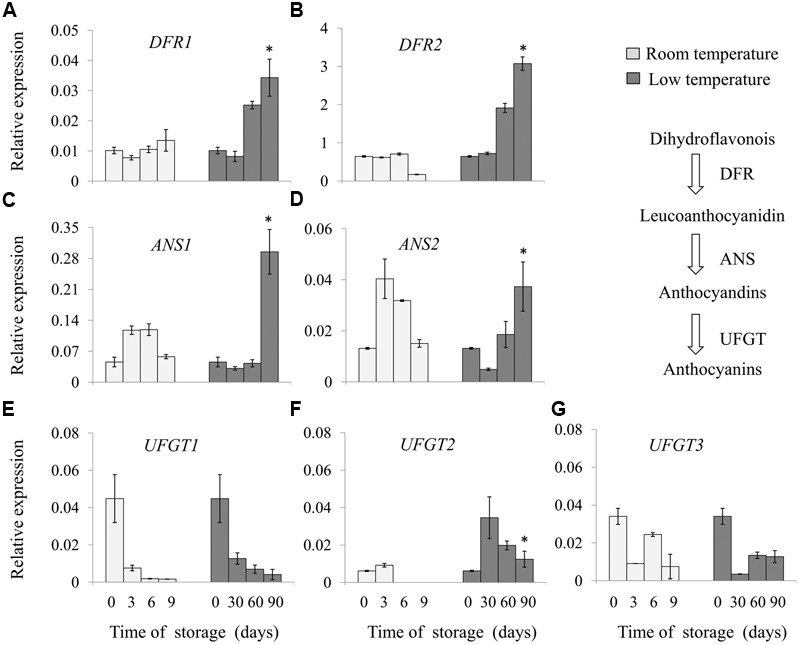
**Relative expression of key genes involved in anthocyanin biosynthesis of ‘Hong yang’ kiwifruit during storage at room temperature for 9 days or low temperature for 90 days, respectively. (A–G)** Show the relative expression of *DFR1, DFR2, ANS1, ANS2, UFGT1, UFGT2*, and *UFGT3*, respectively. Data are means ± SD of three replicates. Error bars represent standard error of means. Asterisk indicates significant differences between fruits stored at room temperature and low temperature at the end of storage according to the Student’s *t*-test at *P* < 0.05.

Full-length cDNA sequence of genes involved in the anthocyanin biosynthesis pathway, were cloned from ripe ‘Hongyang’ kiwifruit using RT-PCR and RACE, annotated as *DFR1* (*Achn014341*), *DFR2* (*Achn0135311*), *ANS1* (*Achn002561*), *ANS2* (*Achn361621*), *UFGT1* (*Achn209671*), *UFGT2* (*Achn017071*) and *UFGT3* (*Achn321621*) according to previous description by Li et al. (2015). Expression level of those genes, i.e., *DFR, ANS*, and *UFGT*, were analyzed in fruit stored at room- and low-temperature (**Figure 2**).

Significant differences in the expression pattern of these genes were revealed during storage. The transcript abundance of *DFR1* and *DFR2* was not changed in fruit stored at room temperature, whereas it was obviously increased in fruit stored at low temperature, with a higher level after 90 days, about 3.4 and 4.8 times that of the initial storage, respectively. *ANS* expression gradually increased in the early stage of storage, with the maximum value at 3 and 6 days, respectively, and then declined in room temperature-stored fruit. By contrast, fruits stored at low temperature remained a lower level of the expression of *ANS1* and *ANS2* in the first 60 days of storage, but exhibited a higher level after 90 days. The expression of *UFGT1* and *UFGT3* decreased in fruit stored either at room temperature or low temperature; however, *UFGT2* expression firstly increased and then declined. Low temperature storage enhanced the *UFGT2* expression from 30 to 90 days. These results indicated that the mRNA levels of specific anthocyanin biosynthesis genes were induced by low temperature.

### Effect of Low Temperature Storage on the Expression of MYB Family Genes

From the draft kiwifruit genome, four full-length MYB-related sequences were cloned from the ‘Hongyang’ kiwifruit, annotated as *MYBA1-1* (*Achn104391*), *MYB4a* (*Achn020361*), *MYB5-1* (*Achn148821*) and *MYB5-2* (*Achn366791*). Phylogenetic analysis using the predicted protein sequences of the four kiwifruit MYBs and other published MYBs sequences suggested that MYB1-1 was most closely related to VvMYBPA1, and MYB4a was clustered with AtMYB4. Moreover, MYB5-1 and MYB5-2 were included in a small group with VvMYB5a and VvMYB5b (Supplementary Figure S2).

**FIGURE 3:**
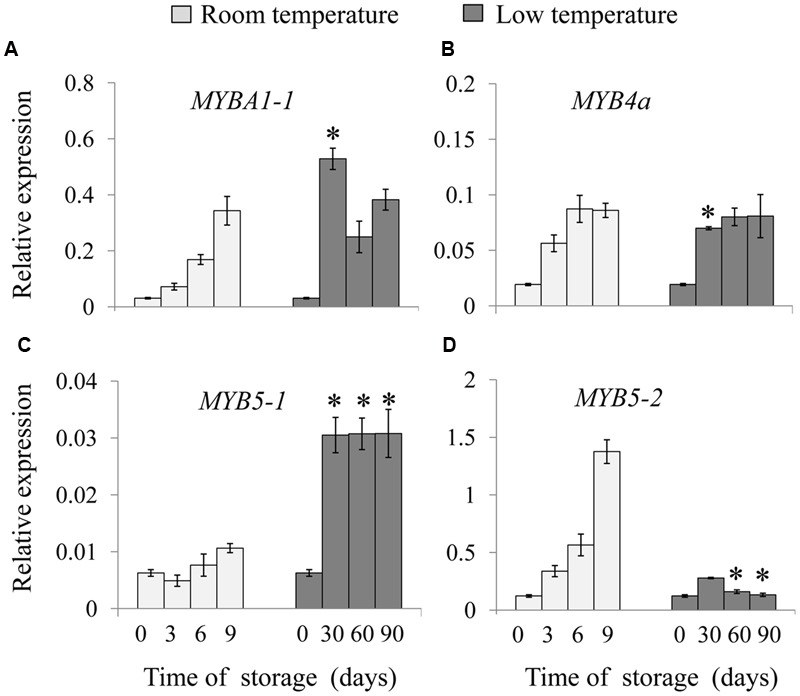
**Relative expression of MYB transcription factors involved in anthocyanin biosynthesis of ‘Hong yang’ kiwifruit during storage at room temperature for 9 days or low temperature for 90 days, respectively. (A–D)** Show the relative expression of *MYBA1-1, MYB4a, MYB5-1*, and *MYB5-2*, respectively. Data are means ± SD of three replicates. Error bars represent standard error of means. Asterisk indicates significant differences between fruits stored at room temperature and low temperature at the paired time points (3 days at 25°C vs. 30 days at 0°C, 6 days at 25°C vs. 60 days at 0°C, 9 days at 25°C vs. 90 days at 0°C) according to the Student’s *t*-test at *P* < 0.05.

Expression levels of the four *MYBs* were analyzed in room and low temperature-stored fruit, respectively. As shown from **Figure 3A**, mRNA levels of *MYBA1-1* increased gradually in room temperature-stored fruit, while its level rapidly increased in low temperature-stored fruit. There was no significant difference in the expression of *MYB4a* during low temperature storage (**Figure 3B**). Interestingly, the most significant differences were observed in the expression patterns of *MYB5-1* and *MYB5-2* in kiwifruit. The expression of *MYB5-1* had a constantly lower level in fruit stored at room temperature throughout the storage time; however, it was sharply increased in low temperature-stored fruit, being a five times higher than that of initial time point, after 90 days of storage (**Figure 3C**). On the contrary, the *MYB5-2* expression in room temperature-stored fruits increased gradually and peaked at 9 days, however, it remained at a basal level till the end of storage in low temperature-stored fruit (**Figure 3D**). The results indicated that low temperature storage could induce and enhance the expression of certain members of *MYB*s gene family.

### Transient Expression in *N. benthamiana*

**FIGURE 4:**
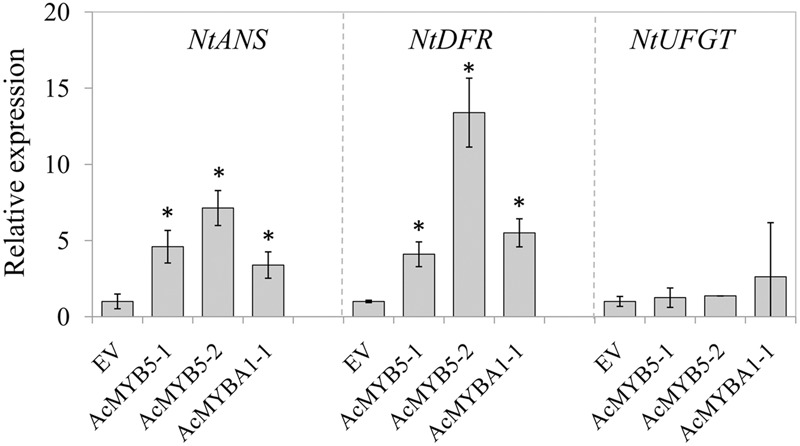
**Gene expression of *NtANS, NtDFR*, and *NtUFGT* in tobacco leaves transiently overexpressing AcMYB5-1/5-2/A1-1.** Data are means ± SD of three replicates. Error bars represent standard error of means. Asterisk indicates significant difference at *P* < 0.05.

To further investigate the roles of AcMYB5-1/5-2/A1-1 in regulation of anthocyanin biosynthesis, transient transformation of genes encoding the three transcription factors was carried out in *Nicotiana benthamiana* leaves. The expression levels of *NtANS, NtDFR*, and *NtUFGT* were analyzed in transiently transformed tobacco leaves (**Figure 4**). Transiently overexpression of AcMYB5-1/5-2/A1-1 up-regulated expression levels of *NtANS* and *NtDFR*, but did not alter the expression of *NtUFGT* in tobacco leaves. A strong induction of the *NtANS* and the *NtDFR* genes was found in AcMYB5-2 agro-infiltrated leaves, about 7 and 13 times higher, respectively, than that in control leaves. The results suggested that overexpression of AcMYB5-1/5-2/A1-1 could activate the gene expression of *NtANS* and *NtDFR* in tobacco.

## Discussion

Anthocyanin is preferentially accumulated in the center of ‘Hongyang’ fruit (**Figure 1A**), which makes it is more attractive for customers. Ripening is considered to be related to anthocyanin production in fruits. A number of reports suggested that there was a positive relation between soluble sugar and anthocyanin contents in fruits, such as bayberry (Shi et al., 2014) and grape berry (Dai et al., 2014). Shi and Xie (2014) considered that glycosylation could modify the stability of anthocyanin in aqueous solution. In the present study, we found that anthocyanin content gradually increased in ‘Hongyang’ kiwifruit along with the increase in SSC and decrease in firmness during storage under both room and low temperatures (**Figure 1**). Comparing with fruit at 0 day, anthocyanin content improved 60 and 100% at the end of storage at 25 and 0°C, respectively. These results indicate that maturity and sugar content are positively related to anthocyanin production in harvested ‘Hongyang’ kiwifruit. Further, we noticed that kiwifruit with similar SSC showed different level of anthocyanin, when they were separately stored at different temperature. For example, anthocyanin content of kiwifruit stored at 0°C (90 days) was significantly higher than those stored at 25°C (9 days), indicating that low temperature can enhance anthocyanin accumulation in ‘Hongyang’ kiwifruit. The similar results were also reported in pear (Zhang et al., 2012) and red orange (Lo Piero et al., 2005).

Low temperature stimulating anthocyanin biosynthesis may contribute to induce the expression of some genes involved in anthocyanin biosynthesis. Lo Piero et al. (2005) considered that long cold-storage strongly induced the expression of ‘late gene’ (i.e., *DFR* and *UFGT*) rather than the ‘early gene’ of the anthocyanin biosynthesis in the red orange fruit. Zhang et al. (2012) showed that low temperature treatment enhanced the expression of the anthocyanin biosynthetic genes, especially *ANS* and *UFGT* in the skin of pear fruit, but did not affect the transcript level of *DFR* gene. Hasegawa et al. (2001) reported that the mRNA level of *DFR* was significantly increased under low temperature condition, indicating the important role of *DFR* in the regulation of anthocyanin biosynthesis. In recent, Man et al. (2015b) found that both ‘early’ and ‘late’ structure genes, including *CHS, CHI, F3H, DFR1, LDOX*, and *F3GT2* showed higher expression in ‘Hongyang’ kiwifruit grown in high altitude with lower temperature compared to low altitude area. Moreover, they also found that expressions of *CHS, CHI, F3H, DFR, LDOX, ANR*, and *FLS* were inhibited at 40°C compared with that at 25°C in harvested ‘Hongyang’ kiwifruit (Man et al., 2015a). These results suggested that temperature might affect both anthocyanin and other branches of flavonoid metabolism in kiwifruit. However, the effect of low temperature on anthocyanin accumulation and involving mechanisms are rarely reported in harvested ‘Hongyang’ kiwifruit. In this study, we found that low temperature enhances anthocyanin accumulation in ‘Hongyang’ kiwifruit via stimulating the expression of *ANS1, ANS2, DFR1, DFR2*, and *UFGT2* genes involved in anthocyanin biosynthesis (**Figure 2**).

It has been widely reported that anthocyanin biosynthesis is regulated by MBW complex (Petroni and Tonelli, 2011). All the three components of MBW complex are important for activating anthocyanin synthesis. Among them, MYBs encoded by multi-gene families with diverse spatial expression domains. The MYBs are often more specific in the genes and pathways they target compared with the bHLH and WD40 components, which may be shared with MBW complexes regulating processes (Albert et al., 2014). Recently, Li et al. (2015) have identified 9 R2R3 MYBs potentially participated in anthocyanin metabolism during fruit development using the transcriptome analysis in ‘Hongyang’ kiwifruit. Among them, MYB5 and MYBA have been proved to be positive activators in the early development of ‘Hongyang’ fruit (7 days after anthesis, DAA), where kiwifruit undergo a temporary accumulation of anthocyanin, as well as later during the fruit development (Li et al., 2015; Man et al., 2015b). The fact that the mRNA abundance of *MYB5* and *MYBA1* in ‘Hongyang’ kiwifruit increased along with anthocyanin accumulation during storage time both at room- and low-temperature, further indicates that the two genes are positively related to anthocyanin production. Moreover, MYB regulators might be a critical factor related to anthocyanin biosynthesis under low temperature (Lai et al., 2011). Some results indicated that *VvMYB5a* and *VvMYB5b* in grape, along with *MdMYBA* in apple, could be induced by low-temperature, then activated the expression of *ANS*, leading to the anthocyanin accumulation in fruit (Ban et al., 2007; Deluc et al., 2008). Our results demonstrated that low temperature enhanced the expression of *MYBA1-1* and *MYB5-1*, but not *MYB5-2* (**Figure 3**), possibly resulting in the induction of specific *ANS, DFR* and *UFGT* expression and thereby the increase of anthocyanin accumulation in ‘Hongyang’ kiwifruit during storage. We also found that the mRNA levels of MYB5-1 and MYB5-2 exhibited opposite trends during storage at different temperatures. It is possible that both MYB5-1 and MYB5-2 have similar function in anthocyanin biosynthesis, but appear to be responsible for different environment factors. Transiently overexpression of AcMYB5-1/5-2/A1-1 in *N. benthamiana* leaves up-regulated expression levels of *NtANS* and *NtDFR*, which further indicate that those MYB transcription factors may participate in anthocyanin biosynthetic pathway. However, we did not observe the changes in leaf color and accumulation of anthocyanins. Transient transformation of MYBs may be not enough to activate the whole biosynthetic pathway of anthocyanins in *N. benthamiana* leaves.

In summary, in the present study, we found that anthocyanins gradually increased in ‘Hongyang’ kiwifruit during post-harvest storage under both room and low temperatures. Low temperature storage could enhance anthocyanin accumulation, and induce the expression of several structural and regulatory genes related to anthocyanin biosynthesis. Other regulators (such as bHLHs and WD40) and involved molecular mechanisms need to be further addressed.

## Author Contributions

ST conceived and designed the experiments. BL and YX performed the experiments. BL, YX, YW, and GQ analyzed the data. BL, YW, and ST drafted the manuscript. All authors read and approved the final manuscript.

## Conflict of Interest Statement

The authors declare that the research was conducted in the absence of any commercial or financial relationships that could be construed as a potential conflict of interest.
