# Supplementary material for: Characterization of Genes Encoding Key Enzymes Involved in Anthocyanin Metabolism of Kiwifruit during Storage Period
Source: Front Plant Sci. 2017 Mar 10;8:341. doi: 10.3389/fpls.2017.00341 (PMC5344892; doi:10.3389/fpls.2017.00341)
Supplement: Supplementary file 1 [file Table_1.PDF]

Supplementary Material

Characterization of genes encoding key enzymes involved in anthocyanin metabolism of kiwifruit during storage period

Boqiang Li[1], Yongxiu Xia[1,2], Yuying Wang[1], Guozheng Qin[1], Shiping Tian[1, 2] *

[1]Key Laboratory of Plant Resources, Institute of Botany, Chinese Academy of Sciences, Beijing 100093, China

[2]University of Chinese Academy of Sciences, Beijing 100049, China

***Correspondence:**

Shiping Tian

Tel.: +86 10 62836559; Fax: +86 10 82594675.

E-mail: tsp@ibcas.ac.cn

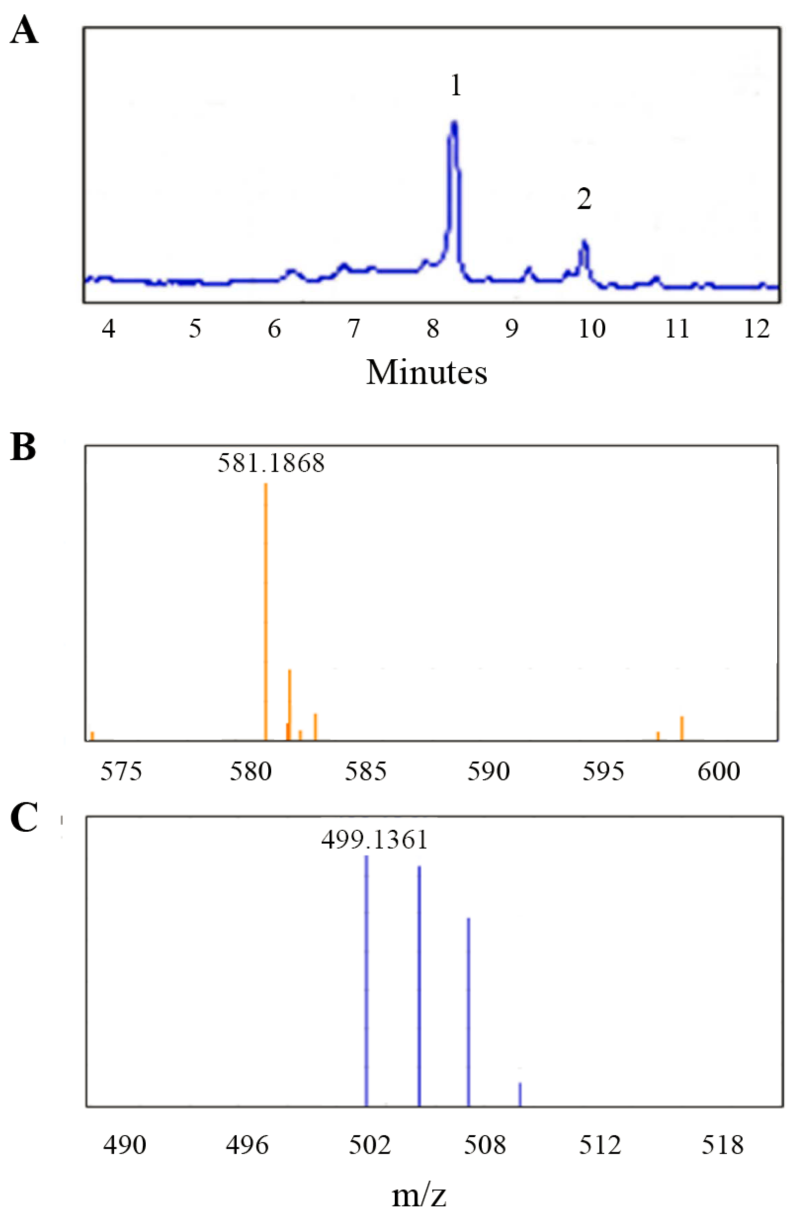

Fig. S1. HPLC and mass spectrum of anthocyanin from ‘Hong yang’ kiwifruit. (A) Representative chromatogram traces (520 nm). Peaks: cyanindin 3-O-xylo(1-2)-galactoside, 1; cyanidin 3-O-galactoside, 2. (B) and (C) mass spectrum of 3-O-xylo(1-2)-galactoside and cyanidin 3-O-galactoside.

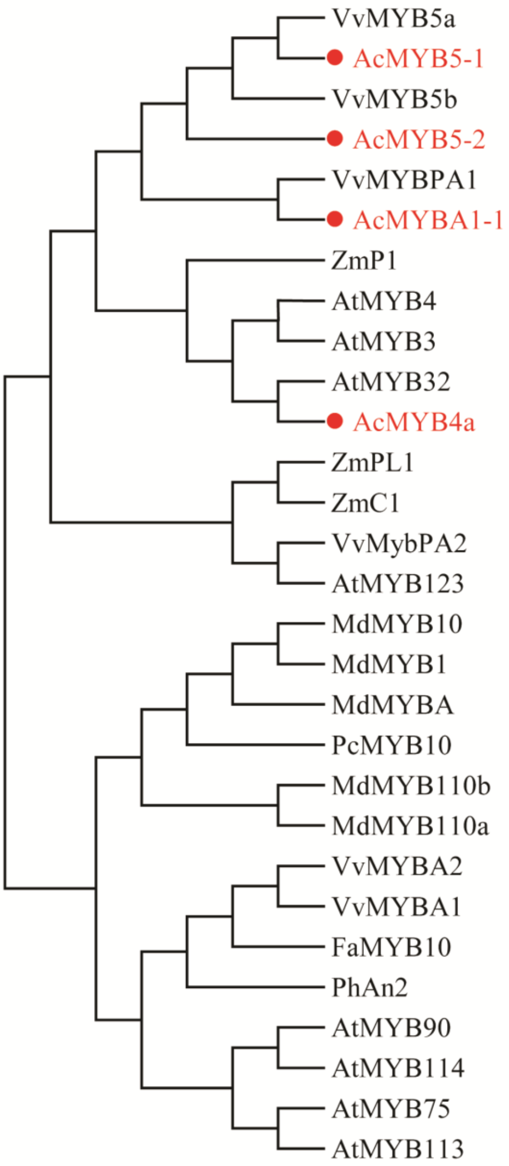

Fig. S2. Phylogenetic analysis of 'Hong yang' kiwifruit MYB protein sequences. Phylogenetic tree was generated using MEGA version 6.0. Bootstrap values from 1,000 replicates for each branch are shown. Origins of the genes are indicated by a prefix as follows: *Ac*, *Actinidia chinensis*; *Vv*, *Vitisvinifera*; *Zm*, *Zeamays*; *At*, *Arabidopsis thaliana*; *Md*, *Malus x domestica*; *Pc*, *Pericalliscruenta*; *Fa*, *Fragaria x ananassa*; *Ph*, *Morusalba*.

Table S1. Primer sequences used in 3' RACE for cloning of *DFR*, *ANS* and *UFGT* genes.

Name	Gene ID[1]	Primers	
		GSP1 (5'-3')	GSP2 (5'-3')
DFR1	Achn014341	CTGTTCGAGCATCCACAAGCC	AGCAAGAAGTACCCCGAGTACGACAT
DFR2	Achn135311	CGCCGGGTTCATCGGTTCTT	GCTATCCAGGGTTGCTCTGGCG
ANS1	Achn002561	CCGGGCCTCCAGCTATTC	CCAGCTATTCTACGAGGGCAAA
ANS2	Achn361621	GGGCTCGTGAACAAGGAAAA	AGCCCCCCAAGGAGAAGAT
UFGT1	Achn209671	AAGGTGGCGGCAAAGGGC	GTGACTTGCCCAGTGGAGTCTTG
UFGT2	Achn017071	ATGCTACACAAAATGGGAC	TCCCTTCAGCCTAATGTCATCATCAT
UFGT3	Achn321621	GGGGGTGCCTTGGGTTCCATAC	CCCACCATCACTGTCATCATCAC

[1]Gene ID, release from the International Kiwifruit Genome Consortium (IKGC).

Table S2. Primers used in the quantitative RT-PCR analysis.

Name	Gene ID[1]	Primers	Sequence (5`-3`)
DFR1	Achn014341	Forward<br>Reverse	CATTTGCTCGTCGCACAGTT<br>CATTTGCTCGTCGCACAGTT
DFR2	Achn135311	Forward<br>Reverse	TTGTTGGCCCTTTCATTATGC<br>ATTGTCCTTGCTTGATGATGGA
ANS1	Achn002561	Forward<br>Reverse	GGGCTCGTGAACAAGGAAAA<br>GTGAATGTGTTGGGCAAAGGT
ANS2	Achn361621	Forward<br>Reverse	TCATGCACATTGGCGATACA<br>AGCGGCTTCAGGATGATCTTC
UFGT1	Achn209671	Forward<br>Reverse	GCCCGTCCAACCTAACATCA<br>CATCAGGTTTCGGTTTTGCTACT
UFGT2	Achn321621	Forward<br>Reverse	TCCCCAATCACAAGTTTTAGCA<br>CTGCACCATTCGGCTATTCA
UFGT3	Achn017071	Forward<br>Reverse	CAAGTTTCGCAAGGTCCTCAA<br>GAATCGGCTTTGTGCTCATCA
MYBA1-1	Achn104391	Forward<br>Reverse	AACAAAAAGAAGAAGAACAATCCTAAGC<br>TTGACATTGAGAAAGAGCCTAAAGAC
MYB4a	Achn020361	Forward<br>Reverse	TCGCATGCAGTTTGGGAATA<br>CCCGGCTTTGCTACTTCCA
MYB5-1	Achn148821	Forward<br>Reverse	TGGTGTTGGATGGGATGCT<br>GGGCTCATGTCGGTTAAAACC
MYB5-2	Achn366791	Forward	TGGTCGTTGATAGCTGGAAGAA

		Reverse	CGATGCCTTGGCTGATGAG
Actin	Achn008281	Forward<br>Reverse	AAGGAAATTACCGCTTTGGC<br>ACATCTGCTGGAAGGTGCTG
NtDFR	EF421429	Forward<br>Reverse	CAAGAGAAATGGCCGGAGTACT<br>GACGAAAAATACACCACGGACAA
NtANS	AB289447	Forward<br>Reverse	CGGAGGCAAGGAGGACCTA<br>CGCCAAGTGCTAGTTCTGGTT
NtUFGT	AB723685	Forward<br>Reverse	GTGCATTGGATGCCTTTTTCA<br>TATTGCTCTTTCTTTTAGCCCTTCA
NtTubA1	AJ421411	Forward<br>Reverse	TGCTTTTGAGCCATCTTCCA<br>CACGGAACATAAGGCAGCAA

[1]Gene ID, release from the International Kiwifruit Genome Consortium (IKGC) or from NCBI GenBank.

Table S3. Primer sequences used for cloning of *AcMYB* genes.

Name	Gene ID[1]	Primers	Sequence (5`-3`)
MYBA1-1	Achn104391	Forward<br>Reverse	ATTTGGAGAGGACAGGGTACCATGAACTATCTA<br>GCTCACCATTCTAGAGGATCCAATCAGCAAAGA
MYB5-1	Achn148821	Forward<br>Reverse	ATTTGGAGAGGACAGGGTACCATGAGGCATCCATCACAG<br>GCTCACCATTCTAGAGGATCCGAACCGCTTCTCAGCATG
MYB5-2	Achn366791	Forward<br>Reverse	ATTTGGAGAGGACAGGGTACCATGAGGCAGCCATCGAGC<br>GCTCACCATTCTAGAGGATCCATTTTGAGCATGATCAAG

[1]Gene ID, release from the International Kiwifruit Genome Consortium (IKGC) or from NCBI GenBank.
